# Synthetic Amphoteric Cryogels as an Antidote against Acute Heavy Metal Poisoning

**DOI:** 10.3390/molecules26247601

**Published:** 2021-12-15

**Authors:** Alzhan Z. Baimenov, Ildar R. Fakhradiyev, Dmitriy A. Berillo, Timur Saliev, Sergey V. Mikhalovsky, Talgat S. Nurgozhin, Vassilis J. Inglezakis

**Affiliations:** 1 National Laboratory Astana, Nazarbayev University, Nur-Sultan 010000, Kazakhstan; 2 Faculty of Chemistry and Chemical Technology, Al-Farabi Kazakh National University, Almaty 050012, Kazakhstan; 3 Department of Pharmaceutical and Toxicological Chemistry, Pharmacognosy and Botany, School of Pharmacy, Kazakh National Medical University, Almaty 050012, Kazakhstan; ildariko@mail.ru (I.R.F.); Berillo.d@kaznmu.kz (D.A.B.); saliev.t@kaznmu.kz (T.S.); tnurgozhin@kaznmu.kz (T.S.N.); 4 Department of Chemisorption and Hybrid Materials, Chuiko Institute of Surface Chemistry, 03164 Kyiv, Ukraine; sergeymikhalovsky@gmail.com; 5 ANAMAD Ltd., Brighton BN1 9SB, UK; 6 Department of Chemical & Process Engineering, University of Strathclyde, Glasgow G1 1XJ, UK

**Keywords:** cryogel, antidote, DMPS, elimination, heavy metals

## Abstract

The effectiveness of an amphoteric cryogel (AAC) as an oral sorbent (enerosorbent) for the treatment of acute poisoning of small animals (rats) with heavy metals (HMs) was studied in in vivo experiments. The morphological structure of the cryogel was examined using scanning electron microscopy/energy-dispersive X-ray analysis and confocal microscopy. The use of the cryogel in the treatment of rats administered an LD_50_ dose of Cd(NO_3_)_2_, CsNO_3_, Sr(NO_3_)_2_, or HgCl_2_ in aqueous solution showed their high survival rate compared to the control group, which did not receive such treatment. The histological and chemical analysis of internal tissues and the biochemical analysis of the blood of the experimental animals showed the effectiveness of the cryogel in protecting the animals against the damaging effect of HMs on the organism comparable with unithiol, a chelating agent based on 2,3-dimercapto-1-propane sulfonic acid sodium salt (DMPS) approved for the treatment of acute poisoning with some heavy metals.

## 1. Introduction

Heavy metals and metalloids (HMs) are omnipresent in the environment in different forms and compositions. Their intake by humans and animals mainly occurs by the ingestion of contaminated food and water. HMs can find their way into the human food chain via soil–plant–water pathways [1]. In contaminated areas, the population can be exposed to chronic HMs intake, which may have a detrimental effect on health, particularly on children and pregnant women. At present, no efficient methods of human health protection against chronic exposure to HMs are available. The suggested treatment modalities relate to acute accidental or suicidal poisoning with HMs [2,3,4,5,6], which is considered to be a rare event and classified as an orphan disease [2]. Even though HMs poisoning is rare, there are cases when it has affected many people. For example, a significant release of radioactive isotopes of Sr and Cs into the environment occurred with the Chernobyl and Fukushima Daiichi nuclear power plant accidents [3,4]. In both cases, large territories in the surrounding areas populated by thousands of inhabitants were contaminated. Globally, thousands of acute and chronic HMs poisoning accidents are reported every year. According to a report of the American Association of Poison Control Centers, more than 9200 people suffered from acute HMs poisoning in 2018 in the US alone [5]. Human exposure to cadmium and mercury can lead to systemic injury of many internal organs such as kidneys, lungs, liver, the reproductive system, and bones [6,7,8]. A fairly significant deposition of HM ions in the tissues of the liver and kidneys is possibly associated with the high mitochondrial activity of these tissues, which leads to the exacerbation of their toxicity [9]. HMs are metabolized in the liver, where they bind to the low molecular weight proteins (<10 kDa)—metallothioneins. These proteins are widespread throughout the organism and contain a large amount of the amino acid cysteine containing the –SH group, which gives them a high affinity for many HM ions and facilitates their accumulation [10]. Oxidative stress is one of the most important mechanisms associated with the toxicity of HMs due to the imbalance between the prooxidant and antioxidant systems, which leads to an excessive amount of reactive oxygen species [11]. For example, cadmium accumulates in mitochondria blocking their respiratory chain in complex III, which further leads to the mitochondrial dysfunction and the formation of free radicals that activate caspase enzymes and the process of apoptosis [10]. Different routes of intake of HM salts can cause different forms of damage to internal organs, among which the oral route is the most detrimental to internal organs, especially the liver [12]. There are few antidotes approved for the treatment of acute HMs poisoning. They are mainly chelating agents prescribed according to the amount of the poison intake and the severity of the patient’s condition. The most common chelating antidotes approved for use in humans have –SH groups in their structure. Dimercaptosuccinic acid (DMSA) and 2,3-dimercapto-1-propane sulfonic acid or its sodium salt (DMPS, unithiol) chelating agents are examples of antidotes approved by the US FDA and/or the European Medicines Agency [13,14]. The chemical rationale of chelation therapy can be explained using the hard and soft acid–base concept, according to which the metal ion (Lewis acid) and the chelator (Lewis base) should have a similar “hardness”, i.e., soft metals (e.g., Pd^2^^+^, Ag^+^, Hg^2+^, Cu^2+^, Cd^2+^, Pt^2+^, and Pb^2^^+^) should be chelated by materials with soft ligands (e.g., DMSA and DMPS), whereas hard metals (e.g., Cr^3+^, Fe^3+^, Be^2+^, Al^3+^) have high affinity with chelators with hard ligands [15]. Intermediate metals (e.g., Fe^2+^, Co^2+^, Ni^2+^, and Zn^2+^) can be chelated by both hard and soft bases. A number of chemical complexes was considered as antidotes in the experiments on acute cadmium and mercury poisoning in animals [13,16,17]. Nevertheless, to date there are no approved antidotes for medical treatment of acute poisoning with a number of HMs such as Cd^2+^, although some -SH containing chelators are currently under experimental development [18]. Generally, HM antidotes are administered parenterally or orally. Oral effectiveness of DMSA and DMPS may be limited due to their low intestinal absorption which is estimated at being 20% for DMSA and 39% for DMPS [19]. Moreover, these agents could facilitate excretion of essential metal ions such as Ca^2+^, Cu^2+^ and Zn^2+^, which results in various abnormalities of physiological functions [20,21]. The reported side-effects of using DMSA and DMPS are the gastrointestinal discomfort, skin reactions, mild neutropenia and elevated liver enzymes [22]. Besides, HMs are redistributed by these agents to potentiate HM toxicity at intracellular sites of the liver and kidneys [7]. Despite the fact that for a long time chelation therapy has been considered the most common method of treating acute heavy metal poisoning [23], there are no approved antidotes for the decorporation of certain radioisotopes from the human body, most notably radiostrontium. A special case is Prussian blue, which has high affinity with thallium and cesium. It has been approved as the antidote against poisoning with these metals including radiocesium but it is not efficient in other HMs removal. Some adsorbents such as pectin, chitosan and activated carbons (AC) were reported as capable of eliminating radioactive isotopes and HMs from the human body [24,25]. After the Chernobyl accident, several pectin- and AC-based adsorbents were tested for removing radiostrontium and radiocesium in animal studies and from the human organism [26]. Although some chelators have a proven ability to remove various heavy metals and radioisotopes in acute cases, the investigation and study of new treatment methods and materials are essential especially for human protection against chronic HMs intake. This is important for the protection of humans chronically exposed to heavy metal pollution and their intake with food and water. To date there are no officially accepted treatments for such people. Although some chelators, such as unithiol, which was used in this study, have been approved for use in the treatment of acute poisoning with certain heavy metals, they cannot be recommended for chronic HM spoisoning treatment because of the likelihood of side effects of the chelating therapy. Most chelators are administered parenterally or intravenously, which requires professional supervision, whereas enterosorption can be performed by the patient. There are hydrogels approved for oral (intestinal) administration, such as polymethylsiloxane polyhydrate Enterosgel^®^, but they have low capacity for HMs retention and are used in the treatment of other conditions [27]. A polymer cryogel enterosorbent containing various functional groups capable of binding different species of HMs could open an opportunity for designing a universal antidote against HM poisoning, suitable for both acute and chronic poisoning treatment. Its unique bimodal porous structure (macropores and nanopores) ensures good adsorption kinetics through macropores and a large adsorption capacity of the nanopores. A single dose of an enterosorbent is usually between 5 and 15 g, which means that a single dose of the cryogel would have a surface area of approximately 600 m^2^, sufficient for binding heavy metal ions in the gastrointestinal tract. Exploring the potential of the polymers and other materials of this type as oral sorbents for human protection against acute and chronic poisoning with HMs merits further long-term studies. It was shown that some polymer cryogels are able to remove high concentrations of mercury, cadmium, cesium, and strontium ions from aqueous media at various pH [28,29,30].

In this study, we evaluated the antidotal effect of a substituted acrylamide-based cryogel (AAC) by assessing its ability to eliminate some toxic metals administered to the small experimental animals (rats) at an LD_50_ dose. To the best of our knowledge, it is the first report on testing a cryogel material as an oral sorbent for HM detoxification in vivo. Its effectiveness was compared with unithiol, the commercial antidote approved for use in Europe for mercury and arsenic poisoning [14,31] and shown promising results in experimental studies for the treatment of some other HMs poisoning [8,16]. 

## 2. Results and Discussion

### 2.1. Synthesis and Characterization of the Polyacrylamide Cryogel

**Figure 1:**
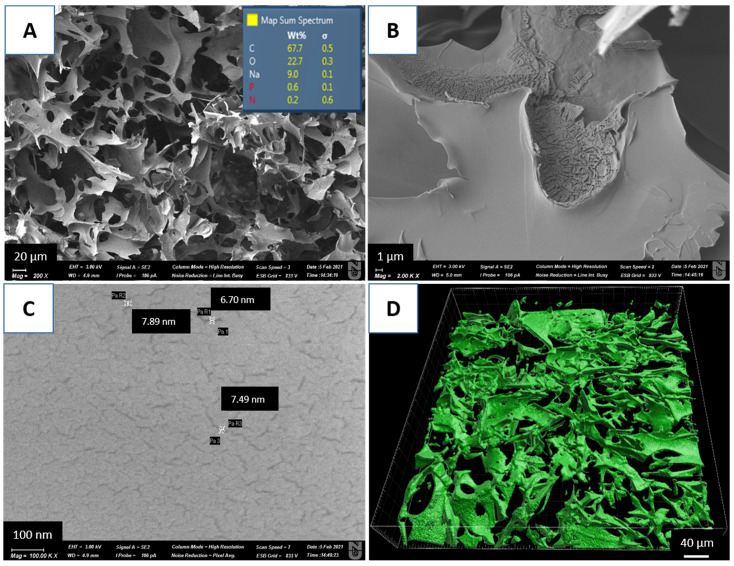
SEM/EDX images of AAC cryogel at magnifications of (**A**) 200×, (**B**) 2000×, and (**C**) 10,000× and (**D**) a confocal microscopy image at 20× magnification.

According to our previous studies, the cryogel used contains carboxylic, amide(I), amide(II), and amine functional groups that are involved in the chelation reaction with metal ions [30]. The SEM image of the cryogel shows a highly developed macroporous structure with a pore size in the range from 10 to 100 μm (Figure 1A). The confocal microscopy image presented in Figure 1D is consistent with the SEM analysis confirming the macroporous structure of the synthesized polymer. SEM/EDX analysis is often used for elemental composition estimation of cryogels [28,32,33]. According to the EDX mapping, the cryogel consists mainly of carbon (68 wt%), oxygen (23 wt%), and sodium (9 wt%) (Figure 1A). Sodium was introduced in the polymer after washing with NaOH. Lower than expected, according to the chemical formula of the polymer, the nitrogen content reflects the fact that the EDX analysis of this element in most materials is unreliable due to the fact of its very weak response [34]. Hydrogen is not detectable by EDX. At higher magnifications, nanopores in the range from 3 to 8 nm were revealed in the cryogel walls (Figure 1B,C). More information and characterizations can be found in our previous publications.

### 2.2. Survival and Clinical Observations of Animals Administered Heavy Metal Salts

After the HM administration, each rat was observed hourly, with clinical signs and mortality being monitored. The type and severity of the signs were recorded individually. After 1 h of heavy metal poisoning, some rats in the untreated Positive Control Group II moved slowly, showed emphatic noise sensitivity and frequent convulsions. Similar cognitive functioning in experimental animals after the introduction of heavy metals has been shown in the literature. The animals treated with enterosorbents in Group III and Group IV and in Negative Control Group I showed normal cognitive functions, and no adverse signs of anxiety, antisociality, and motor dysfunctions were observed. 

#### 2.2.1. General Signs and Behavioral Analysis

The animals were observed for obvious behavioral, neurological, and toxic effects within 24 h. The toxicological effects of HMs were assessed by the mortality rate expressed as an average lethal dose (LD_50_) value (Table 1).

#### 2.2.2. Survival Rate of Rats in the Experimental Groups

**Figure 2:**
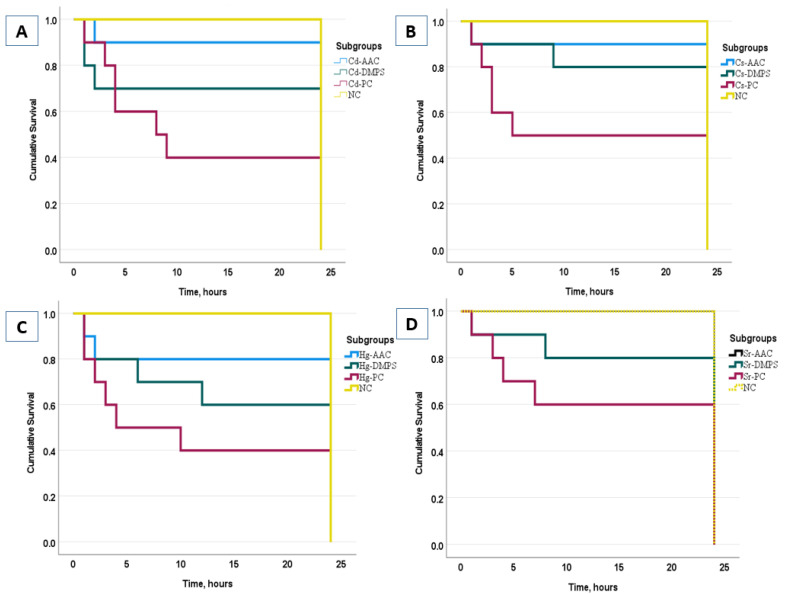
Plots of the Kaplan–Meier product limit estimates of the survival of animals for the Cd (**A**), Cs (**B**), Hg (**C**), and Sr (**D**) subgroups.

The animals in Group II (Positive Control) poisoned with cadmium and mercury salts died within 2–4 h after their administration, while untreated animals in the subgroups that received strontium and cesium salts died within 4–7 h. Most fatal cases in Groups III and IV treated with the AAC cryogel or unithiol occurred 15–18 h after the metal intake. The survival rates were calculated using the Kaplan–Meier method. The Kaplan–Meier survival curve is defined as the probability of surviving in a given length of time while considering time in many small intervals [35]. Herein, the survival of each animal in each subgroup during the 24 h of the experiment was evaluated. The survival functions of the Kaplan–Meier plots and statistical data, which evaluated the cumulative survival rate of animals in each group during 24 h of experiment, are presented in Figure 2. 

The survival analysis showed that the NC group did not significantly differ from the AAC and DMPS groups (*p* ≥ 0.05), while comparing the results between the AAC and DMPS groups, it can be noted that, in general, the survival rate in the AAC group was higher than in the DMPS group (*p* ≤ 0.05). As expected, the lowest survival rate was found in the PC group compared to the NC group (*p* ≤ 0.001).

#### 2.2.3. Histopathological Studies of Liver and Kidneys

**Figure 3:**
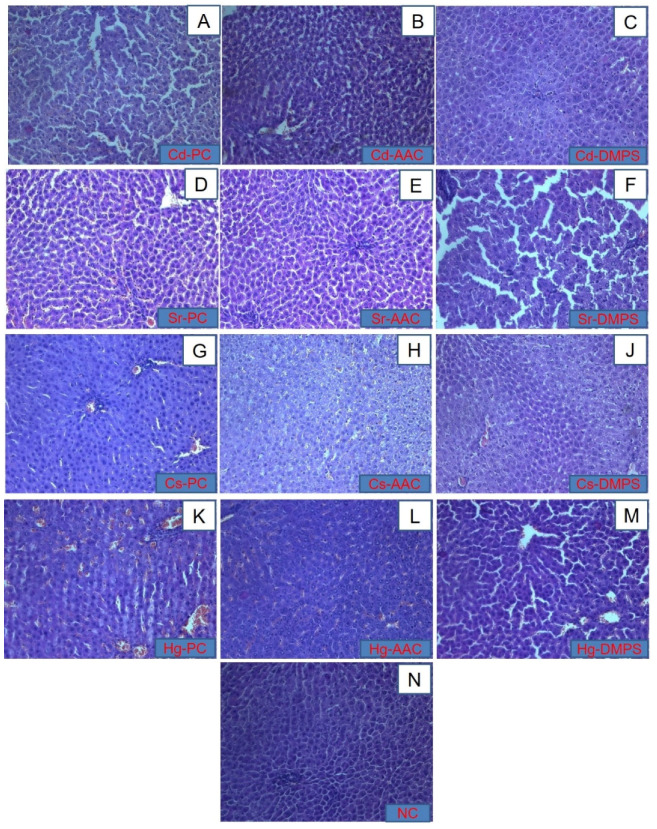
Histological images of liver from (**A**) Cd-PC, (**B**) Cd-AAC, (**C**) Cd-DMPS, (**D**) Sr-AAC (**E**) Sr-AAC (**F**) Sr- DMPS, (**G**) Cs-PC (**H**) Cs-AAC (**J**) Cs-DMPS (**K**) Hg-PC (**L**) Hg-AAC (**M**) Hg-DMPS subgroups, and (**N**) NC group, 100× magnification, H&E staining.

Histopathological images of liver tissues of the experimental animals are presented in Figure 3A–M. Histopathological analysis of the liver of the control group of animals demonstrated the typical structure of the organ (Figure 3N). During the morphological study of the histological parameters of the liver of animals poisoned with cadmium and strontium (Figure 3A,D), it was found that the introduction of metals in a dose of LD_50_ led to a significant expansion of sinusoidal capillaries and central veins. The introduction of a more massive dose of the toxicant caused a change in the architectonics of the hepatic lobules, a violation of the traditional orientation of hepatocyte beams. The morphological study of the liver in experimental animals administered mercury or cesium revealed pronounced hydropic and fatty degeneration of hepatocytes with focal necrosis, and profuse hemorrhage in sinusoidal capillaries (Figure 3G,K). The histological examination of the liver of animals from the Cd-AAC subgroup (Figure 3B) showed a slight hemorrhage in the sinusoid capillaries and damage to hepatocyte cells, while the sample Cd-DMPS (Figure 3C) showed an almost intact liver structure with a slight hemorrhage in the intercellular space. However, when studying liver samples after the administration of strontium followed by the introduction of antidotes, a directly opposite picture was observed. The liver of the sample Sr-AAC (Figure 3E) looked intact without visible damage, while the liver of the Sr-DMPS (Figure 3F) animal subgroup was severely damaged with marked increases in sinusoidal capillaries and dystrophic changes in cells. In the case of the cesium poisoning treatment, the liver samples of Cs-AAC and Cs-DMPS (Figure 3H,J, respectively) looked slightly damaged with a minor hemorrhage and deformation of some hepatocytes. Apparently, these results are associated with the fact that the negative effect of Cs on the liver appears only after prolonged exposure to Cs [36], since no significant pathological changes were observed in the Cs-PC control group (Figure 3G). An extremely high degree of liver damage was observed in the Hg-DMPS sample (Figure 3M); it was characterized by acute venous hyperemia of the liver, hemorrhage, and necrosis of hepatocytes in the central zone of the hepatic lobule. The sample Hg-AAC (Figure 3L) exhibited less damage with slight hemorrhage.

**Figure 4:**
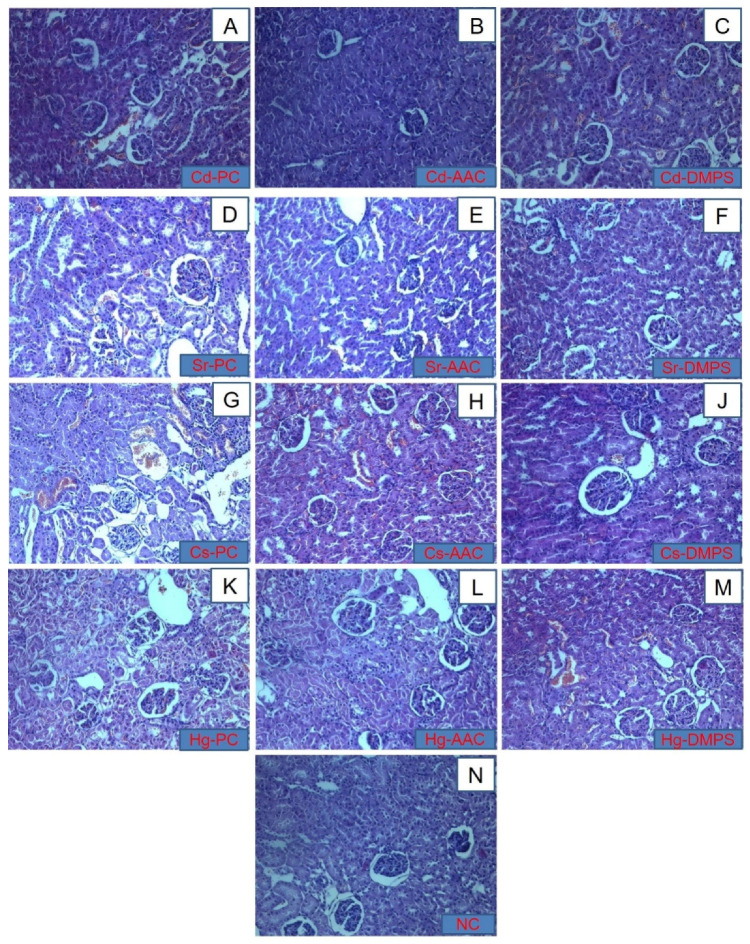
Histological images of kidneys of (**A**) Cd-PC, (**B**) Cd-AAC, (**C**) Cd-DMPS, (**D**) Sr-AAC, (**E**) Sr-AAC, (**F**) Sr-DMPS, (**G**) Cs-PC, (**H**) Cs-AAC, (**J**) Cs-DMPS, (**K**) Hg-PC, (**L**) Hg-AAC, and (**M**) Hg-DMPS subgroups and the (**N**) NC group at 100× magnification, H&E staining.

The histopathological images of kidneys of healthy, poisoned, and treated animals are presented in Figure 4. In healthy animals (Group I), the capsule, cortical, and brain layers were microscopically distinguishable (Figure 4N). Numerous glomeruli of nephrons with a spherical shape and a slightly uneven surface were clearly visible in the cortical layer. The glomeruli were enclosed in capsules, the lumen of which was sickle-shaped or surrounded the glomerulus in the form of a ring. The cavity of the capsule was free of contents. The space between the glomeruli was represented by a homogeneously colored fabric with numerous rounded sections of convoluted tubules and vessels of the cortical substance. The tubule epithelium adhered strictly to the surface of the basement membrane, represented by a continuous unicellular layer of endothelial cells. The nuclei of these cells were rounded, regular in shape with a smooth surface. They were located in the center of a homogeneous cytoplasm. The lumen of the tubules was gaping and free of content. The brain substance was represented by homogeneously colored parenchyma with a parallel tubular apparatus.

In the Positive Control Group II of animals poisoned with metals, significant changes in the structure were observed in the cortical substance. In the lumens of the capsules of most glomeruli, there were amorphous deposits and red blood cells (Figure 4A,D,G,K). In the tubules of all levels, significant dystrophic changes in the epithelium were noted, which were manifested in the swelling and separation of epithelial cells from the basement membranes and from each other, the loss of a significant number of basophilia nuclei, karyolysis, and desquamation of epithelial cells. Gleams of tubules were significantly expanded. In the interstitium, a pronounced edema, lymphocytic, and neutrophilic infiltration was observed and had a focal character. The cortical vasculature looked diluted and filled with blood.

In animals treated with the cryogel (Group III), the lumen of individual capsules contained amorphous inclusions (Figure 4B,E,H,L). Although the tubular apparatus contained signs of dystrophic changes, they were much less pronounced than in untreated Group II. In contrast to the control group, in which tubular obstruction by amorphous masses was observed throughout the thickness of the section, in this group, these changes were focal in nature and were localized mainly in the zone of transition of cortical substance to the brain. The degree of desquamation of the epithelium and pathological changes in the nuclei was also significantly less pronounced than in the control. In animals treated with unithiol (Group IV), (Figure 4C,F,J,M) the lumen of individual capsules in histological sections contained amorphous inclusions and showed a moderate degree of congestion in renal blood vessels and atrophy of some glomeruli. The signs of dystrophic changes in the tubular apparatus were significantly lesser than in Group II but slightly higher than in the cryogel-treated Group III.

The results of the histological analysis revealed that, in terms of preventing the development of pathological changes in the liver and kidneys caused by the intake of strontium, cesium, and mercury, the cryogel enterosorption resulted in a more positive histological picture with minimization of the development of irreversible dystrophic changes compared to PC Group II and the unithiol enterosorption (Group IV). However, with regard to the mitigation of cadmium intake, the antidotal effect of unithiol enterosorption was more pronounced in comparison with the cryogel. It is worth noting that both sorbents were administered only once, one hour after the intake of heavy metals. This fact indicates that the effectiveness of the enterosorbents in emergency cases of heavy metal poisoning could be potentially enhanced by their more frequent administration. In terms of reducing the deposition of cadmium, cesium, and mercury ions in the internal organs, DMPS/unithiol enterosorption showed higher efficiency than AAC. Previously published studies also reported the effectiveness of DMPS in faster urinary excretion of mercury through the kidneys [37]. Oral absorption of water soluble DMPS was approximately 39%, being the reason for its usually intravenous rather than oral administration. It is rapidly metabolized into a disulfide form [18]. Ionized forms of toxic heavy metals are present in acute intoxication. Due to the fact of their properties that form a complex or conjugate in the blood, the concentration of free ionized forms was less than 10% of the total metal concentration [38].

#### 2.2.4. Effect of HM Poisoning and Enterosorption Treatment on Biochemical Markers

Blood biochemical parameters provide essential information for determining the damage to the liver and kidneys. The levels of biochemical blood markers in the experimental animal groups are presented in Table 2. Following the metal intake, the enzymatic activity of AST, ALT, GGT, and alkaline phosphatase in the blood serum tended to increase, but in Group III of the cryogel-treated animals, these parameters returned to normal, except for alkaline phosphatase. In Group IV (unithiol treated), the ALT levels remained within normal limits, and AST was higher than in the NC Group I and Group III treated with cryogels but lower than in the metal-poisoned Group II.

The TP level in the Sr-DMPS (70.9 ± 3.7 g/L) and Hg-DMPS (64.1 ± 3.4 g/L) subgroups was statistically significantly higher in comparison with the NC group (60.35 ± 2.3 g/L) (*p* ≤ 0.01). Compared with the control group (4.19 ± 0.2 mM), there was a statistically significant increase in the level of urea in the Sr-AAC (10.4 ± 5.0 mM) and Cs-DMPS (22.7 ± 9.1 mM) (*p* ≤ 0.01) subgroups as well as in the Sr-PC subgroup (7.8 ± 2.9 mM) (*p* ≤ 0.05). Glu in the Cd-PC, Sr-PC, Cs-DMPS, and Hg-DMPS subgroups was statistically significantly higher (*p* ≤ 0.05) as well as in the Sr-DMPS subgroup (*p* ≤ 0.001) than in the NC Group I (11.9 ± 1.0 mM). In comparison with the NC group (53.5 ± 8.4 U/L), all subgroups of Group II had high ALT levels, which was regarded as a statistically significant difference (*p* ≤ 0.01). AST indices with a significance level equal to *p* ≤ 0.01 in the Sr-PC and Hg-PC groups as well as with a significance *p* ≤ 0.05 in the Cd-PC and Cs-PC groups statistically significantly increased in comparison with the control group indices (91.8 ± 9.5 U/L). In addition, in contrast to the NC group without intervention (0.81 ± 0.27 U/L), a statistically significant increase in the GGT level was observed in the Cd-PC and Sr-PC groups (*p* ≤ 0.05) as well as in the Hg-PC, Sr-DMPS, and Cs-DMPS (*p* ≤ 0.01) groups. ALP indices with a significance level of *p* ≤ 0.05 in groups Cd-PC, Sr-PC, Cs-PC, Cd-AAC, Sr-AAC, Hg-AAC, and Hg-DMPS as well as with a significance equal to *p* ≤ 0.01 in the Sr-DMPS and Cs-DMPS groups were higher compared to the ALP level in the NC group, which was 115.5 ± 23.3 U/L. In contrast to the TC level of the group of intact animals (1.02 ± 0.20 µM), the TC level was higher in the blood of animals of the experimental groups Sr-PC (2.06 ± 0.73 µM) and Sr-DMPS (2.25 ± 0.19 µM), and the level of statistical significance was *p* ≤ 0.05 and *p* ≤ 0.01, respectively. In general, the results of biochemical blood marker analysis showed that the levels of all parameters of animals treated with the cryogel and unithiol remained within normal limits and corresponded to the negative controls, whereas the positive controls showed elevated values, which indicates the destruction of the liver and kidneys of the poisoned animals. Our findings are similar to other studies that have reported an increase in blood biochemical markers due to the fact of heavy metal intoxication [39,40,41]. Thus, it can be concluded that both antidotes, amphoteric cryogel and unithiol, are effective sorbents and can reduce the negative effect of heavy metal intoxication.

#### 2.2.5. Evaluation of the Heavy Metal Content in Animal Tissues

The results of the evaluation of HM presence in various tissues of the poisoned and treated animals are shown in Table 3. No metals were detected in the control group. Enterosorption in both Group III and Group IV led to a statistically significant reduction in Cd levels in all tissues studied in comparison to the PC Group II. DMPS eliminated cadmium more effectively than the cryogel; the content of Cd was three times lower in the stomach and two times lower in the duodenum of unithiol treated animals compared to cryogel-treated animals. When studying the effectiveness of cryogel and unithiol against strontium, it was found that both antidotes had similar metal-binding activity that could reduce the metal content in tissues 3–5-fold. Despite the use of antidotes, the levels of cesium in all organs of the studied subgroups were consistently high, except for the stomach in which both the cryogel and unithiol reduced the cesium concentration two-fold and omentum in which the use of enterosorbents reduced cesium content by 30% (cryogel) and two-fold (unithiol) compared to the Cs-PC subgroup. Since the initial concentration LD_50_ of the cesium dose was very high (2390 mg/kg/bw), neither cryogel nor unithiol could adsorb all cesium ions, the non-adsorbed ions of which were found in high concentrations in the animals’ organs. In the PC subgroup of animals poisoned with mercury, high concentrations of mercury were observed in all studied tissues. After the treatment with unithiol, the mercury content in all tissues was very low, whereas the cryogel enterosorption showed a statistically significant decrease in the mercury content only in the liver and omentum compared with the positive control.

However, in the renal tissue and the intestinal tissue (duodenum) of the Cs-DMPS subgroup and in the liver of the Cs-AAC subgroup, an increase in the concentration of cesium was noted after enterosorption in contrast to the Cs-PC group (*p* ≤ 0.05). 

#### 2.2.6. Study Limitations

In this study, we examined the effectiveness of the cryogel enterosorption in the treatment of acute intoxication with high doses of heavy metal salts. For assessing longer treatment outcomes, it will be necessary to conduct long-term experimental studies simulating the chronic phase of poisoning in which changes in weight, animal well-being, cognitive functions as well as other laboratory indicators of the studied groups of animals will be monitored.

## 3. Materials and Methods

### 3.1. Materials

The reagents, such as N,N-dimethylacrylamide (DMAAm, 99%), allylamine (AA, 98%), methacrylic acid (MAAc, 99%), cross-linking agent N,N-methylenebis(acrylamide) (BisAAm, 99%), 70% H_3_PO_4_, 5M NaOH, ammonium peroxodisulfate (APS, 98%), and *N*,*N*,*N*’,*N*’-tetramethyl ethylenediamine (TEMED, ≥99.5%) were used for the cryogel synthesis. The analytical purity grade of the heavy metal salts Cd(NO_3_)_2_ (98%), CsNO_3_ (99.9%), HgCl_2_ (>99.9%), and Sr(NO_3_)_2_ (>99%) were used in animal studies. All reagents were obtained from Sigma–Aldrich (Darmstadt, Germany) and used as received. The unithiol antidote (ELLARA LLC, Pokrov, Russia) was purchased from the local pharmacy. The water used for the preparation of the acrylamide cryogel (AAC) was purified using a Puris MR-RO1600 (Mirae ST, Anyang, South Korea) reverse osmosis unit, while saline was used for making aqueous HM solutions. 

#### 3.1.1. Synthesis of the Polyacrylamide Cryogel (AAC)

The procedure for the cryogel synthesis has been discussed in detail elsewhere [28,32]. Water was degassed by purging N_2_ for 30 min and used in further steps. Briefly, 0.2186 g of BisAAm were dissolved in 10 mL of water under vigorous stirring followed by adding 0.4125 mL of MAAc and additional acid neutralization by 1.2 mL of 5M NaOH. In another beaker, monomers dimethylacrylamide (DMAAm) (0.3445 g) and AA (0.3 mL) were dissolved in 7.3 mL of water under continuous stirring and acidified with 137 L of concentrated H_3_PO_4_ to convert allylamine into salt. Subsequently, after mixing these two separately prepared solutions together and proper degassing, 15.5 μL of TEMED were added dropwise, mixed, and cooled down to 2–4 °C for 30 min under the nitrogen atmosphere followed by the addition of 0.25 mL 5 wt% of APS at continuous stirring. Finally, 2 mL of the monomeric mixture was poured into plastic syringes with 0.9 cm inner diameter and immersed in the ethanol-cooled cryobath (Julabo F34, Seelbach, Germany) at −12 °C for 24 h. The monolithic cryogels thus obtained were thawed and washed with 10% ethanol and then with 2 L of pure water. For further characterization and experiments, the cryogel samples were lyophilized using a FreeZone 2.5 L (Labconco, Kansas City, MO, USA) freeze-dryer at −54 °C under vacuum (0.5 mbar) for 48 h to remove water. 

#### 3.1.2. Cryogel Characterization

The morphological characteristics of the cryogel were studied using a Zeiss Crossbeam 540 scanning electron microscope (SEM) at 20 kV, equipped with a backscattered electron detector. The samples were coated with a 5 nm gold layer. The cryogel structure was also examined using a confocal laser scanning microscope LSM 780 (Zeiss, Oberkochen, Germany). For better visualization, the polymer discs were immersed in 0.5 M solution of Rhodamine B for 24 h and then washed with water to remove the unbound dye from the cryogel pores. 

### 3.2. In Vivo Animal Studies 

All animal experiments were conducted in the Laboratory of Experimental and Clinical Pharmacology of the Asfendiyarov Kazakh National Medical University (KazNMU) under the permission of the Local Ethics Committee of KazNMU (reg. №643/26.09.2018).

The study was conducted in accordance with the Guide for the Care and Use of Laboratory Animals [42]. During the study, we made every effort to minimize animal suffering and reduce their number.

#### 3.2.1. Experimental Animals

Seven–eight-week-old male mongrel rats (*n* = 130) weighing 210 ± 20 g were purchased and kept in the vivarium at the Kazakh National Medical University (Almaty, Kazakhstan). The rats were housed separately in laboratory animal cages at 22–25 °C with 50–55% humidity and a 12 h light/dark cycle according to the Guide for the Care and Use of Laboratory Animals [43]. The rats were fed a standard diet and allowed access to distilled water ad libitum.

#### 3.2.2. Acute Oral Toxicity

Modeling of acute heavy metal poisoning was carried out using solutions of Cd(NO_3_)_2_, CsNO_3_, Sr(NO_3_)_2_ and HgCl_2_ in saline. Although the data for the LD_50_ doses have been reported in literature for HgCl_2_ [31,44], Cd(NO_3_)_2_ [43,45], CsNO_3_ [46,47] and Sr(NO_3_)_2_ [48], there are significant variations between them which motivated us to determine LD_50_ in this work. 

The study for determining LD_50_ was performed according to the Organization for Economic Co-operation and Development (OECD) Guidelines [49]. In the Guidelines, protocol animals are dosed one at a time. If the animal survives, the dose for the next animal is increased; if the animal dies, the dose for the next animal is reduced. 

The animals were randomly divided into three experimental groups, each of them comprising four subgroups (*n* = 10), according to the type of HM administered and treatment modality, and one negative control group (*n* = 10) (Table 4). The negative control (NC) Group I was fed a standard rat diet without any HM salt addition. The positive control (PC) Group II had the subgroups: Cd-PC, Sr-PC, Cs-PC, and Hg-PC, indicating the metal salt administered; animals in this group did not receive any treatment. In Group III, the animals were administered a HM salt and treated with the AAC enterosorbent at a dose of 250 mg/kg body weight. They were divided into subgroups according to the metal administered: Cd-AAC, Sr-AAC, Cs-AAC, and Hg-AAC. In Group IV, the animals were administered a HM salt and treated with unithiol, the commercial name of DMPS, at the same dose of 250 mg/kg body weight. They were divided into subgroups according to the metal administered: Cd-DMPS, Sr-DMPS, Cs-DMPS, and Hg-DMPS. To model the acute poisoning with HM, the LD_50_ dose of metal, recalculated for each animal weight, was administered by an atraumatic intragastric probe. 

#### 3.2.3. Treatment of Animals by Enterosorption

The animals given an LD_50_ of a heavy metal were treated by enterosorption with AAC cryogel or unithiol. The cryogel was administered after 60 min of HM intake by the oral route through an atraumatic probe in the form of a suspension in doses of 250 mg/kg in 15 mL saline. For the preparation of the cryogel suspension, the samples were ground in an agate mortar into a fine powder with a particle size less than 300 μm. The commercial chelating agent unithiol (purchased from a pharmacy) was used in the same dose dissolved in water (1 mL of solution contained 50 mg of DMPS). The effectiveness of enterosorption was estimated by the 24 h survival rate. After 24 h, the rats were euthanized, and the internal organs (i.e., liver, kidney, stomach, omentum, and duodenum) were withdrawn for further analysis. Laboratory animals were withdrawn from the experiment by the method of cervical dislocation by an experienced researcher very quickly, and no rats showed clinical signs of suffering before they died. The disposal of animals and biological material was carried out in accordance with the procedure for disposal and destruction of biological waste, subparagraph 46-11 of Article 8 of the Law of the Republic of Kazakhstan dated July 10 2002 “On Veterinary Medicine”.

#### 3.2.4. Histopathological Examination

The preserved liver and kidneys of the animals were subjected to a histological examination. The tissue samples were fixed in 10% neutral formalin and then embedded in paraffin followed by preparation of sections with a thickness of 5 μm. A section of each tissue was stained with hematoxylin (H) and eosin (E). The study of the slices was carried out in transmitted light using a Zeiss MR color high-resolution camera of the LSM 780 confocal scanning microscope (Carl Zeiss, Oberkochen, Germany).

#### 3.2.5. Blood Biochemistry Analysis

For the analysis of blood biochemical parameters, the biomarkers, including total serum protein (TP), urea nitrogen (BUN), fasting glucose (GLU), serum aspartate aminotransferase (AST), serum alanine aminotransferase (ALT), total bilirubin (TB), direct bilirubin (BD), gamma-glutamyltransferase (GGT), alkaline phosphatase (ALP), total cholesterol (TC), high-density lipoprotein (HDL), and low-density lipoprotein (LDL), were measured on the biochemical analyzer Cobas Integra 400 plus (Roche Diagnostics, Rotkreuz, Switzerland). 

#### 3.2.6. Microwave Digestion of Tissues

The HM (Sr, Cs, and Cd) content in the animal tissues was measured with the Multiwave Pro (Anton Paar, Graz, Austria) microwave digester. The lyophilized internal organs were weighed and put into 16HF100 rotor vessels containing 5 mL HNO_3_ and 2.5 mL H_2_O_2_. The power-controlled method of tissue digestion was used, and the experimental conditions are presented in Table 5. After digestion, the tissue-containing solutions were filtered through paper filters (Whatman^®^ qualitative filter paper, Grade 1), diluted with water to 25 mL and kept for further studied on AAnalyst 400 (Perkin Elmer, Waltham, MA, USA) atomic absorption spectroscopy (AAS) for metal amount quantification. 

### 3.3. Statistical Analysis

SPSS 22.0 for Windows was used for the statistical analysis. The arithmetic mean (M) and standard deviation (SD) were calculated for quantitative variables. Data were presented as the M ± SD. Qualitative attributes were described as absolute (*n*) and relative (%) values. The calculation of the sample size of laboratory animals was carried out according to the previously described method [35] using the G Power program [50]. The statistical significance analysis was performed by the analysis of variance (one-way ANOVA) for multiple group comparisons or *t*-tests for two group comparisons. Differences were considered statistically significant at *p* ≤ 0.05 (*) and *p* ≤ 0.001 (**).

## 4. Conclusions

The present study showed that intake of high doses of heavy metal salts (Cd, Sr, Cs, and Hg) lead to significant abnormal changes in liver and kidney function of experimental animals, disrupting the regulatory function of liver and kidney enzymes. The intragastric administration of the amphoteric cryogel suspension one hour after poisoning with LD_50_ doses of metals increased the survival rate of the animals at 24 h of the experiment. The histopathological study of the kidneys and liver revealed a marked improvement in the structure of the internal organ tissues. The biochemical blood markers of experimental animals treated with both enterosorbents, AAC, and unithiol improved compared with the Positive Control Group, which did not receive any treatment. After enterosorption, the metal content in the animals’ internal organs mostly decreased with exception of the cesium content in the liver (AAC), kidneys, and duodenum (unithiol). 

In overall, the preliminary results on the use of amphoteric cryogel as an oral antidote in treating acute heavy metal poisoning and preventing its damaging effect on an organism are promising. 

## Figures and Tables

**Table 1:** An average lethal dose (LD_50_) of various heavy metal salts administered orally in experimental rats.

HM Salt	LD_50,_ mg/kg Body Weight
Cd(NO_3_)_2_	300
CsNO_3_	2390
Sr(NO_3_)_2_	1980
HgCl_2_	75

**Table 2:** Effect of heavy metals on the blood biochemical markers in the experimental animals.

	TP, g/L	BUN, mM	Glu, mM	ALT, U/L	AST, U/L	TBil, µM	GGT, U/L	ALP, U/L	TC, µM
**NC**	60.3 ± 2.3	4.19 ± 0.2	11.9 ± 1.0	53.5 ± 8.4	92 ± 9.5	0.9 ± 0.28	0.8 ± 0.27	115 ± 23.3	1.02 ± 0.20
**Cd-PC**	55.9 ± 2.1	5.7 ± 0.6	18.0 ± 3.8 *	139 ± 10.3 **	130 ± 33.5 *	0.8 ± 0.26	2.3 ± 1.5 *	181 ± 80.6 *	0.81 ± 0.52
**Sr-PC**	64.8 ± 3.8	7.8 ± 2.9 *	14.7 ± 1.3 *	137 ± 9.5 **	143 ± 14.4 **	1.05 ± 0.07	2.6 ± 1.34 *	207 ± 64.3 *	2.0 ± 0.73 *
**Cs-PC**	63.1 ± 3.0	6.3 ± 1.6	11.1 ± 1.5	131.4 ± 4.0 **	130 ± 25.7 *	1.1 ± 0.35	1.4 ± 0.71	204 ± 47.4 *	0.59 ± 0.05
**Hg-PC**	59.9 ± 3.8	4.1 ± 0.5	13.7 ± 1.4	194 ± 9.5 **	153 ± 14.4 **	1.1 ± 0.21	4.5 ± 5.02 **	152 ± 17.7	1.05 ± 0.08
**Cd-AAC**	60.8 ± 3.8	7.0 ± 1.2	11.8 ± 0.6	27.3 ± 8.5 *	71 ± 9.5	0.75 ± 0.07	1.2 ± 0.28	219 ± 36.1 *	1.1 ± 0.21
**Sr-AAC**	63 ± 8.8	10.4 ± 5.0 **	10.9 ± 1.9	43.3 ± 1.9	85 ± 7.0	0.9 ± 0.21	0.8 ± 0.0	199 ± 25.5 *	1.2 ± 0.49
**Cs-AAC**	62 ± 8.6	6.2 ± 1.3	10.2 ± 2.0	30 ± 12.8	93 ± 8.3	1.3 ± 0.21	0.5 ± 0.77	178 ± 101.8	0.77 ± 0.48
**Hg-AAC**	57.2 ± 2.7	6.1 ± 0.1	11.1 ± 2.8	39.5 ± 6.6	89 ± 14.8	0.9 ± 0.35	0.4 ± 0.21	200 ± 16.3 *	1.05 ± 0.25
**Cd-DMPS**	59.4 ± 3.0	6.4 ± 1.8	12.0 ± 2.4	33 ± 12.0	126 ± 42.3 *	1.1 ± 0.49	0.5 ± 0.57	129.5 ± 3.5	0.68 ± 0.35
**Sr-DMPS**	70.9 ± 3.7 **	5.4 ± 0.6	23.2 ± 2.4 **	28.5 ± 6.1	102 ± 23.4	1.3 ± 0.07	3.7 ± 4.67 **	348 ± 57.3 **	2.25 ± 0.19 **
**Cs-DMPS**	55.3 ± 4.5	22 ± 9.1 **	14.0 ± 0.1 *	41± 17.9	133 ± 33.4 *	0.9 ± 0.35	3 ± 3.75 **	358 ± 44.5 **	1.03 ± 0.58
**Hg-DMPS**	64.1 ± 3.4 **	6.8 ± 0.4	15.3 ± 5.9 *	38 ± 7.9	103 ± 3.9	1.3 ± 0.14	0.7 ± 0.92	181 ± 46.7 *	1.13 ± 0.09

* *p* ≤ 0.05 and ** *p* ≤ 0.01 compared with the NC. TP—total protein; BUN—blood urea nitrogen; Glu—glucose; ALT—alanine aminotransferase; AST—aspartate aminotransferase; TBil—total bilirubin; GGT—gamma-glutamyl-transferase; ALP—alkaline phosphatase; TC—total cholesterol.

**Table 3:** Concentration of metal ions in the various tissues of experimental animals.

	Liver, mg/g	Kidney, mg/g	Stomach, mg/g	Omentum, mg/g	Duodenum, mg/g
**NC Group I**	n/d ^&^	n/d	n/d	n/d	n/d
**Cd-PC**	0.234	0.146	8.998	2.498	6.812
**Cd-AAC**	0.105 *	0.035 *	0.899 **	0.104 **	0.202 **
**Cd-DMPS**	0.079 **	0.045 *	0.313 **	0.092 **	0.111 **
**Sr-PC**	0.025	0.065	0.298	0.123	0.150
**Sr-AAC**	0.022	0.038 *	0.103 *	0.032 *	0.037 *
**Sr-DMPS**	0.013 *	0.024 *	0.086 *	0.037 *	0.038 *
**Cs-PC**	10.52	9.79	16.27	13.59	10.63
**Cs-AAC**	14.38 *	13.15	8.18 *	9.81 *	10.16
**Cs-DMPS**	13.07	14.56 *	8.82 *	6.78 **	14.08 *
**Hg-PC**	0.247	0.613	3.035	0.493	0.664
**Hg-AAC**	0.130 *	0.564	2.126	0.112 *	0.420
**Hg-DMPS**	0.014 **	0.030 **	0.207 **	0.046 **	0.040 **

^&^ Not detected. * *p* ≤ 0.05 and ** *p* ≤ 0.01 in comparison with the Positive Control Group II.

**Table 4:** Groups of experimental animals.

Group	Subgroup (*n* = 10 Each)	Metal Salt, Dose + Antidote
Group I (*n* = 10)	NC	negative control, no HM
Group II (*n* = 40) Positive control, no antidote	Cd-PC	LD_50_ Cd(NO_3_)_2_
	Sr-PC	LD_50_ Sr(NO_3_)_2_
	Cs-PC	LD_50_ CsNO_3_
	Hg-PC	LD_50_ HgCl_2_
Group III (*n* = 40)	Cd-AAC	LD_50_ Cd(NO_3_)_2_ + AAC
	Sr-AAC	LD_50_ Sr(NO_3_)_2_ + AAC
	Cs-AAC	LD_50_ CsNO_3_ + AAC
	Hg-AAC	LD_50_ HgCl_2_ + AAC
Group IV (*n* = 40)	Cd-DMPS	LD_50_ Cd(NO_3_)_2_ + unithiol
	Sr-DMPS	LD_50_ Sr(NO_3_)_2_ + unithiol
	Cs-DMPS	LD_50_ CsNO_3_ + unithiol
	Hg-DMPS	LD_50_ HgCl_2_ + unithiol

**Table 5:** The conditions of animal tissues microwave digestion.

Step	Temperature	Power	Time
Power ramp	not controlled	600 W	15 min
Power hold	not controlled	600 W	20 min
Cooling	→ 70 °C	0 W	30 min

## Data Availability

The data presented in this study are available on request from the corresponding author. The data are not publicly available due to the ethical issues.

## Sample Availability

Samples of the cryogels are available from the authors.

## References

[1] Kumar M., Gogoi A., Mukherjee S. (2020). Metal removal, partitioning and phase distributions in the wastewater and sludge: Performance evaluation of conventional, upflow anaerobic sludge blanket and downflow hanging sponge treatment systems. J. Clean. Prod..

[2] (1983). Orphan Drug Act.

[3] Mu W., Yu Q., Li X., Wei H., Jian Y. (2017). Niobate nanofibers for simultaneous adsorptive removal of radioactive strontium and iodine from aqueous solution. J. Alloys Compd..

[4] Moore J.J., Raine T.P., Jenkins A., Livens F.R., Law K.A., Morris K., Law G.T.W., Yeates S.G. (2019). Decontamination of caesium and strontium from stainless steel surfaces using hydrogels. React. Funct. Polym..

[5] Gummin D.D., Mowry J.B., Spyker D.A., Brooks D.E., Beuhler M.C., Rivers L.J., Hashem H.A., Ryan M.L. (2019). 2018 Annual Report of the American Association of Poison Control Centers’ National Poison Data System (NPDS): 36th Annual Report. Clin. Toxicol..

[6] Braitberg G. (2017). Drugs and Antidotes in Acute Intoxication. Critical Care Nephrology.

[7] Rana M.N., Tangpong J., Rahman M.M. (2018). Toxicodynamics of Lead, Cadmium, Mercury and Arsenic- induced kidney toxicity and treatment strategy: A mini review. Toxicol. Rep..

[8] Andersen O. (2016). Chelation Treatment During Acute and Chronic Metal Overexposures—Experimental and Clinical Studies. Chelation Therapy in the Treatment of Metal Intoxication.

[9] Aschner M., Connor J.R., Dorman D.C., Malecki E.A., Vrana K.E., Massaro E.J. (2002). Manganese in Health and Disease. Handbook of Neurotoxicology: Volume I.

[10] Sabath E., Robles-Osorio M.L. (2012). Renal health and the environment: Heavy metal nephrotoxicity. Nefrologia.

[11] Hejazy M., Koohi M.K. (2017). Effects of Nano-zinc on Biochemical Parameters in Cadmium-Exposed Rats. Biol. Trace Elem. Res..

[12] Mouro V.G.S., Siman V.A., da Silva J., Dias F.C.R., Damasceno E.M., do Cupertino M., de Melo F.C.S.A., da Matta S.L.P. (2020). Cadmium-Induced Testicular Toxicity in Mice: Subacute and Subchronic Route-Dependent Effects. Biol. Trace Elem. Res..

[13] Domingo J.L. (1995). Prevention by chelating agents of metal-induced developmental toxicity. Reprod. Toxicol..

[14] Bjørklund G., Mutter J., Aaseth J. (2017). Metal chelators and neurotoxicity: Lead, mercury, and arsenic. Arch. Toxicol..

[15] Flora S.J.S., Pachauri V. (2010). Chelation in metal intoxication. Int. J. Environ. Res. Public Health.

[16] Bernhoft R. (2012). Mercury Toxicity and Treatment: A Review of the Literature. J. Environ. Public Health.

[17] Andersen O. (1989). Choice of chelating antidotes for acute cadmium intoxication. Toxicol. Environ. Chem..

[18] Luczak M.W., Zhitkovich A. (2013). Role of direct reactivity with metals in chemoprotection by N-acetylcysteine against chromium(VI), cadmium(II), and cobalt(II). Free Radic. Biol. Med..

[19] Sears M.E. (2013). Chelation: Harnessing and Enhancing Heavy Metal Detoxification—A Review. Sci. World J..

[20] Torres-Alanís O., Garza-Ocañas L., Bernal M.A., Piñeyro-López A. (2000). Urinary excretion of trace elements in humans after sodium 2,3-dimercaptopropane-1-sulfonate challenge test. J. Toxicol. Clin. Toxicol..

[21] Adams J.B., Baral M., Geis E., Mitchell J., Ingram J., Hensley A., Zappia I., Newmark S., Gehn E., Rubin R.A. (2009). Safety and efficacy of oral DMSA therapy for children with autism spectrum disorders: Part A--medical results. BMC Clin. Pharmacol..

[22] Mehta A., Flora S.J. (2001). Possible role of metal redistribution, hepatotoxicity and oxidative stress in chelating agents induced hepatic and renal metallothionein in rats. Food Chem. Toxicol..

[23] Kim J.-J., Kim Y.-S., Kumar V. (2019). Heavy metal toxicity: An update of chelating therapeutic strategies. J. Trace Elem. Med. Biol..

[24] Kartel M.T., Kupchik L.A., Veisov B.K. (1999). Evaluation of pectin binding of heavy metal ions in aqueous solutions. Chemosphere.

[25] Gutnikova A.R., Mavlyan-Hodjaev R.S., Ismailova M.G., Ashurova D.D., Makhmudov K.O., Saidkhanov B.A. (2010). Assessment of efficiency of different enterosorbents in the correction of the morphological disturbances in the liver and kidney of rats caused by heavy metals salts. Pharm. Her. (Vestnik Farmatsii Russ.).

[26] Nesterenko A.V., Nesterenko V.B., Yablokov A. (2009). V Chapter IV. Radiation protection after the Chernobyl catastrophe. Ann. N. Y. Acad. Sci..

[27] Howell C.A., Mikhalovsky S.V., Markaryan E.N., Khovanov A.V. (2019). Investigation of the adsorption capacity of the enterosorbent Enterosgel for a range of bacterial toxins, bile acids and pharmaceutical drugs. Sci. Rep..

[28] Baimenov A., Berillo D., Abylgazina L., Poulopoulos S.G., Inglezakis V.J. (2018). Novel Amphoteric Cryogels for Cd2+ Ions Removal from Aqueous Solutions. Proceedings of the Key Engineering Materials.

[29] Baimenov A.Z., Berillo D.A., Moustakas K., Inglezakis V.J. (2020). Efficient removal of mercury (II) from water by use of cryogels and comparison to commercial adsorbents under environmentally relevant conditions. J. Hazard. Mater..

[30] Baimenov A., Berillo D., Azat S., Nurgozhin T., Inglezakis V. (2020). Removal of cd^2+^ from water by use of super-macroporous cryogels and comparison to commercial adsorbents. Polymers.

[31] Ansar S., Alghosoon H. (2016). Effect of Diallylsulphide Supplementation on Wistar Rats Exposed to Mercuric Chloride. Trop. J. Pharm. Res..

[32] Baimenov A.Z., Berillo D.A., Inglezakis V.J. (2019). Cryogel-based Ag°/Ag_2_O nanocomposites for iodide removal from water. J. Mol. Liq..

[33] Berillo D. (2020). Gold nanoparticles incorporated into cryogel walls for efficient nitrophenol conversion. J. Clean. Prod..

[34] Liao Y. (2006). Practical Electron Microscopy and Database. www.globalsino.com/EM/.

[35] Festing M.F.W., Altman D.G. (2002). Guidelines for the design and statistical analysis of experiments using laboratory animals. ILAR J..

[36] Souidi M., Tissandie E., Grandcolas L., Grison S., Paquet F., Voisin P., Aigueperse J., Gourmelon P., Guéguen Y. (2006). Chronic contamination with 137cesium in rat: Effect on liver cholesterol metabolism. Int. J. Toxicol..

[37] Cao Y., Skaug M.A., Andersen O., Aaseth J. (2015). Chelation therapy in intoxications with mercury, lead and copper. J. Trace Elem. Med. Biol..

[38] Barbier O., Jacquillet G., Tauc M., Cougnon M., Poujeol P. (2005). Effect of heavy metals on, and handling by, the kidney. Nephron. Physiol..

[39] Wang X., Wang B., Zhou M., Xiao L., Xu T., Yang S., Nie X., Xie L., Yu L., Mu G. (2021). Systemic inflammation mediates the association of heavy metal exposures with liver injury: A study in general Chinese urban adults. J. Hazard. Mater..

[40] Franciscato C., Moraes-Silva L., Duarte F.A., Oliveira C.S., Ineu R.P., Flores E.M.M., Dressler V.L., Peixoto N.C., Pereira M.E. (2011). Delayed biochemical changes induced by mercury intoxication are prevented by zinc pre-exposure. Ecotoxicol. Environ. Saf..

[41] Peixoto N.C., Serafim M.A., Flores E.M.M., Bebianno M.J., Pereira M.E. (2007). Metallothionein, zinc, and mercury levels in tissues of young rats exposed to zinc and subsequently to mercury. Life Sci..

[42] (2011). Guide for the Care and Use of Laboratory Animals.

[43] Dote E., Dote T., Shimizu H., Shimbo Y., Fujihara M., Kono K. (2007). Acute lethal toxicity, hyperkalemia associated with renal injury and hepatic damage after intravenous administration of cadmium nitrate in rats. J. Occup. Health.

[44] Mahour K., Saxena P.N. (2009). Assessment of haematotoxic potential of mercuric chloride in rat. J. Environ. Biol..

[45] Yakuji G. Pharmaceuticals Monthly. https//www.cdc.gov/niosh/idlh/7440439.html.

[46] Tarasenko N.I., Lemeshevskaia E.P. (1978). Effect of cesium compounds on the body. Vestn. Akad. Med. Nauk SSSR.

[47] Cochran K.W., Doull J., Mazur M., Dubois K.P. (1950). Acute toxicity of zirconium, columbium, strontium, lanthanum, cesium, tantalum and yttrium. Arch. Ind. Hyg. Occup. Med..

[48] Tarasenko N.I., Lemeshevskaia E.P. (1976). Experimental basis of the maximum permissible concentrations of strontium compounds in the air of industrial premises. Materialy eksperimental’nogo obosnovaniia predel’no dopustimykh kontsentratsii soedinenii strontsiia v vozdukhe proizvodstvennykh pomesc. Gig. i Sanit..

[49] (2008). Test, No.425: Acute Oral Toxicity: Up-and-Down Procedure.

[50] Faul F., Erdfelder E., Lang A.-G., Buchner A. (2007). G*Power 3: A flexible statistical power analysis program for the social, behavioral, and biomedical sciences. Behav. Res. Methods.

